# The integration of GPS and visual navigation for autonomous navigation of an Ackerman steering mobile robot in cotton fields

**DOI:** 10.3389/frobt.2024.1359887

**Published:** 2024-04-12

**Authors:** Canicius Mwitta, Glen C. Rains

**Affiliations:** ^1^ College of Engineering, University of Georgia, Athens, GA, United States; ^2^ Department of Entomology, University of Georgia, Tifton, GA, United States

**Keywords:** dynamic window approach, fully convolutional neural network, precision agriculture, pure pursuit, path between cotton row detection

## Abstract

Autonomous navigation in agricultural fields presents a unique challenge due to the unpredictable outdoor environment. Various approaches have been explored to tackle this task, each with its own set of challenges. These include GPS guidance, which faces availability issues and struggles to avoid obstacles, and vision guidance techniques, which are sensitive to changes in light, weeds, and crop growth. This study proposes a novel idea that combining GPS and visual navigation offers an optimal solution for autonomous navigation in agricultural fields. Three solutions for autonomous navigation in cotton fields were developed and evaluated. The first solution utilized a path tracking algorithm, Pure Pursuit, to follow GPS coordinates and guide a mobile robot. It achieved an average lateral deviation of 8.3 cm from the pre-recorded path. The second solution employed a deep learning model, specifically a fully convolutional neural network for semantic segmentation, to detect paths between cotton rows. The mobile rover then navigated using the Dynamic Window Approach (DWA) path planning algorithm, achieving an average lateral deviation of 4.8 cm from the desired path. Finally, the two solutions were integrated for a more practical approach. GPS served as a global planner to map the field, while the deep learning model and DWA acted as a local planner for navigation and real-time decision-making. This integrated solution enabled the robot to navigate between cotton rows with an average lateral distance error of 9.5 cm, offering a more practical method for autonomous navigation in cotton fields.

## 1 Introduction

The demand for increased production at a reduced cost in agriculture has necessitated the need for automation. Automation in agriculture improves farming efficiency, enhances safety, reduces costs, and reduces the need for human labor, ultimately leading to an increase in productivity. To achieve this, mobile robotic systems have been introduced in agricultural fields to automate tasks such as weeding, harvesting, spraying, scouting, planting, and monitoring. Driving these machines is a very demanding task, which involves steering to follow a path while operating the equipment (Kise et al., 2005; Heraud and Lange, 2009). Steering automation is a crucial step towards autonomous vehicles which allows more precise coordination between navigation in the field and the performance of the main operation such as harvesting, weeding, or other tasks. Autonomous driving in the agricultural industry has seen significant breakthroughs recently, but compared to self-driving cars, the agricultural environment is significantly different in terms of complexity and diversity (Binbin et al., 2021). Most autonomous vehicles in agriculture utilize the Global Navigation Satellite System (GNSS) for navigation (Shalal et al., 2013; Gao et al., 2018). They determine their absolute position by utilizing real-time kinematic global position system (RTK-GPS) data and navigate by following a path formed from a series of pre-recorded GPS coordinates (Stoll and Kutzbach, 2000; Bakker et al., 2010; Khan et al., 2018; Fue et al., 2020; Moeller et al., 2020). Studies have shown GPS guidance can be effective (Stoll and Kutzbach, 2000; Bakker et al., 2010; Khan et al., 2018; Fue et al., 2020; Moeller et al., 2020); however, weather, obstacles, and satellite availability can affect its performance. Furthermore, without visual or ranging sensor, GPS navigation can be vulnerable to collisions in the field since it lacks obstacle detection.

Alternative solutions for autonomous navigation in agricultural fields have emerged with advances in computer vision technologies. Traditional computer vision algorithms have been used to detect either crop rows or the space between them as the path. Studies have utilized monocular RGB cameras to capture the scene and use computer vision algorithms to segment the images for crop row detection and path finding (Rovira-Más et al., 2005; Ji and Qi, 2011), while others have used stereo vision to get a three-dimensional (3D) field image for crop row detection (Kise et al., 2005). Moreover, vision-based sensors like laser range finders which use LIDAR (Light Detection and Ranging) have been used by studies such as Hiremath et al. (2014) and Higuti et al. (2019) to follow crop rows in agricultural fields. Traditional computer vision techniques do not require extensive computational resources; however, they are sensitive to changes in illumination, and occlusion which can be exaggerated in outdoor agricultural environments. Additionally, color-based segmentation techniques are sensitive to weeds between crop rows, and most do not handle different crop growth stages well. A reliable autonomous navigation system in agriculture would need to be robust against the challenging conditions in the field, such as changes in illumination, weather, occlusion, weed presence, and crop growth stages. Advances in deep learning technology have provided powerful and robust methods for distinguishing between crop rows and paths between rows. This is done through training deep learning models with extensive examples of images of different environmental scenery to improve future predictions. Many recent studies have utilized deep learning models as part of their autonomous navigation system in agriculture (Adhikari et al., 2020; Aghi et al., 2020; Bah et al., 2020; Cerrato et al., 2021; de Silva et al., 2021; Doha et al., 2021). For example, Adhikari et al. (2020) used a limited dataset and deep learning to develop a neural network that is robust against shadows, crop growth stages, and row spacing, however, it failed to generalize well to areas where crops had occluded the path. Bah et al. (2020) developed a solution that combined convolutional neural network (CNN) and Hough transform to achieve crop row detection in UAV captured images, which was robust to weeds in the field.

Advances in road lane marking detection for self-driving vehicles have inspired studies which use deep learning based semantic segmentation. For example, Adhikari et al. (2020), de Silva et al. (2021), and Doha et al. (2021) utilized fully convolutional neural network (FCN) for semantic segmentation (Long et al., 2015), known as ‘U-net’ because of its ‘U-like’ structure (Ronneberger et al., 2015), and some computer vision algorithms to detect crop rows in agricultural fields. U-Net is a popular image segmentation algorithm known for not demanding a lot of data for training and having low latency in prediction. Most of these models were robust against shadows and row discontinuities but still struggled with changes in light conditions and presence of weeds.

After determining the paths’ locations, the autonomous system needs to navigate by following the path through path planning and tracking. Different implementations of path planning and tracking have been deployed in agricultural settings to enable navigating the predicted paths. Solutions like pure pursuit path tracking (Fue et al., 2020), Non-linear model predictive path tracking (Backman et al., 2012), sliding mode control for non-linear tracking (Tu et al., 2019), sliding window approach for path planning (Adhikari et al., 2020), genetic algorithms (Noguchi and Terao, 1997), and others have been implemented.

As the cost of farming rises rapidly due to labor shortages (Guthman, 2017; Richards, 2018; Zahniser et al., 2018), the need for autonomous navigation in agricultural fields becomes increasingly crucial, especially in the context of precision agriculture. This study hypothesized that a combination of GPS and visual navigation will be effective in autonomously navigating a mobile robot in an agricultural field. To do this, the main objectives that were achieved were to investigate GPS navigation, visual navigation, and the combination of both. The paper explored using and combining GPS, and FCN for semantic segmentation, while utilizing other computer vision algorithms to autonomously navigate a mobile robot in a cotton field. The study also explored two path-planning and tracking algorithms, pure-pursuit (Coulter, 1992), and dynamic window approach (DWA) (Fox et al., 1997), on their effectiveness to follow the desired path while autonomously navigating the robot. Instead of detecting the crop rows, the FCN for semantic segmentation in this study concentrated on detecting the paths between the rows. The faster predictions feature of the model enables real-time path detection and planning for weeding, harvesting, and scouting in cotton. Moreover, to generate a path between cotton rows on the ground plane that the rover can follow, the detected path must be mapped from the image domain to ground coordinates. This study proposes a method for achieving that requirement.

## 2 Materials and methods

### 2.1 Platform

**FIGURE 1:**
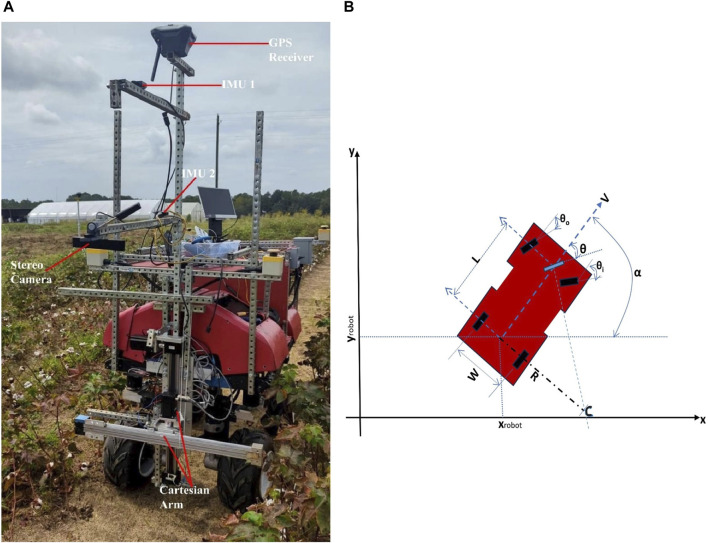
**(A)**The robotic platform, **(B)** Kinematic model of Ackerman Steering robot mechanism. ( $x_{robot}$ ***;*** $y_{robot}$ ) are the coordinates of the rear axle midpoint, ∝ is the robot orientation, θ is the steering angle, and L is the wheelbase.

The robotic platform used in this study was a 4-wheel Ackerman (car-like) steering ground rover (see Figure 1A). The robot navigates between rows of cotton using various sensors, actuators, microcontrollers, and an embedded computer. Each of the rover’s wheels is run by a 250 W Pride Mobility wheelchair motor (Pride Mobility Products Corporation, Duryea, PA, United States of America), with the two back wheels connected to Quadrature rotary encoders (CUI AMT 102 (CUI Devices, Lake Oswego, Oregon, United States of America)) to provide feedback on wheel rotation. The motors are driven by two Cytron MDDS30 motor controllers (Cytron Technologies, Pulau Pinang, Malaysia) and powered by two 20,000 mAh 6-cell Tunigy LIPO batteries (Turnigy, kwun Tong, Hong Kong). A linear servo (HDA8-50) (ServoCity, Winfield, Kansas, United States of America) is connected to the front wheels for steering. To track the rover’s orientation two PhidgetSpatial Precision 3/3/3 High Resolution Inertia Measurement Units (IMUs) (Phidgets Inc, Calgary, Canada) are mounted to the rover. These modules contain a 3-axis accelerometer, a 3-axis gyroscope, and a 3-axis compass. An Arduino Mega microcontroller (Arduino, Italy) controls these sensors and actuators. A single-band EMLID Reach RS + RTK GNSS receiver (Emlid, Budapest, Hungary) is mounted to the rover for GPS navigation, while a Zed2 stereo camera (Stereo Labs, San Francisco, California, United States) is utilized for visual navigation. The stereo camera has two image sensors which allow it to capture normal RGB images, calculate depth of pixels in the image, and generate 3D point cloud. The rover also has a 2D Cartesian arm that is used for various in-field tasks like harvesting, and weeding. The autonomous navigation system is run by an embedded computer (Nvidia Jetson Xavier AGX with 8-core ARM v8.2 64-bit CPU, 32 GB of RAM, and a 512-core Volta GPU (Nvidia Corporation, Santa Clara, California, United States)) which communicates with other components using Robotic Operating System (Quigley et al., 2009) (ROS 1–Noetic). ROS is a set of software frameworks for robot software implementation which provides processes presented as nodes in graph structure that are connected by edges known as topics. Topics carry and pass messages between nodes.

The RTK-GNSS receiver obtained corrections from another RTK GNSS receiver (EMLID Reach RS 2) which was set up as a base station at a fixed absolute position in the field. With base correction the rover can achieve centimeter-level precise position.

### 2.2 Kinematics modeling of the robot

The robot uses front-wheel Ackerman steering mechanism (see Figure 1B) which was designed to solve sideways tire slipping when following a curved path. The XY plane represents the ground plane where the robot navigates. The center of rotation (C) is on the line extended from the rear axle intersecting the axes of the front wheels. When steering, the front inside wheel must turn a greater angle ( $\theta_{i}$ ) than the outside wheel ( $\theta_{o}$ ). The robot position is taken at the midpoint of the rear axle ( $x_{robot}$ ***;*** $y_{robot}$ ).

The kinematics model considers the middle of the rear wheels as the robot’s position where, $R\;\left(m\right)$ is the turning radius of the robot, $L\;\left(m\right)$ is the wheelbase (distance between rear and front wheels), $W\;\left(m\right)$ is the width of the robot, $V\;\left(\frac{m}{s}\right)$ is the linear velocity of the robot, $\propto \left(rad\right)$ is the heading, and $\theta \;\left(rad\right)$ ideal front wheel turning angle. The relationships are defined as follows:

$$\tan\left(\theta_{i}\right)=\frac{2Ltan\left(\theta \right)}{2L-Wtan\left(\theta \right)}$$

$$\;\;\tan\left(\theta_{o}\right)=\frac{2Ltan\left(\theta \right)}{2L+Wtan\left(\theta \right)}$$

$${\dot{X}}_{robot}=Vcos\left(\propto \right)$$

$${\dot{Y}}_{robot}=Vsin\left(\propto \right)$$

$$\;\;\dot{\propto }=\frac{V}{L}\tan\;\left(\theta \right)$$

Where ${\dot{X}}_{robot}\;\left(\frac{m}{s}\right)$ and ${\dot{Y}}_{robot}\;\left(\frac{m}{s}\right)$ are the horizontal and vertical components of the rover’s velocity V and $\dot{\propto }$ (rad/s) is the angular velocity of the rover.

### 2.3 GPS navigation

#### 2.3.1 Sensor fusion using Extended Kalman Filter

Extended Kalman Filter (EKF) is an algorithm that efficiently estimates the internal state of a non-linear dynamic system from a series of noisy measurements. Derived from Kalman filter (Kalman, 1960) which can only estimate linear systems, EKF is a well-known non-linear state estimator which has been implemented in many studies (Smith et al., 1962; Wan and Nelson, 2001; Moore and Stouch, 2016). Our goal is to accurately estimate the pose and velocity of the robot over time by fusing multiple noisy sensors. Considering, the robot’s state (pose) at time t , $x_{t}$ , f as a non-linear transition function, $w_{t}$ the process noise (normally distributed), $z_{t}$ , the measurement received from sensors at time t , h as a non-linear sensor model, $v_{t}$ the measurement noise, and $u_{t}$ as the control, the process and measurements can be described with two equations.

$$\;\;x_{t}=f\left(x_{t-1},u_{t}\right)+w_{t}$$

$$z_{t}=h\left(x_{t}\right)+v_{t}$$

The time step, ∆t depends on the speed of updating the filter. The filter was updated at a frequency of 20 Hz, which implies a time step of 50 ms.

**FIGURE 2:**
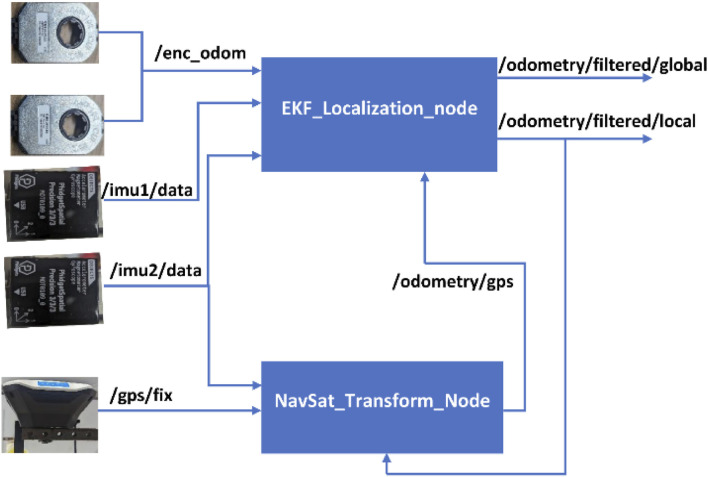
ROS–Robot_localization package implementation with odometry topics output from different sensors input topics.

The EKF implementation fused continuous data from encoders, IMUs and GPS using the ROS package Robot_localization (Moore and Stouch, 2016). Robot_localization package accepts position, linear velocity, angular velocity, linear acceleration, and angular acceleration data from sensors and then estimates the robot’s pose and velocity. Two nodes; a state estimation node EKF_localization_node and a sensor processing node NavSat_Transform node (see Figure 2) implemented in Robot_Localization were fed sensor data to estimate the local and global pose and velocity of the robot. Data from two IMU topics (*/imu1/data* and*/imu2/data*) fused with the odometry topic (*/enc_odom*) obtained the two encoder readings and kinetic modeling equations were fused by the EKF_localization_node to get locally accurate odometry estimates in the topic*/odometry/filtered/local*. The NavSat_Transform_Node transformed geographic coordinates (latitude, longitude) into robot’s world frame and produced GPS odometry topic*/odometry/gps* from fusing data from GPS topic*/gps/fix*, IMU topic*/imu2/data*, and the local estimate from EKF_Localization_node/odometry/filtered/local. The GPS odometry*/odometry/gps* was then fed to EKF_Localization_node to obtain an accurate and complete global state (*/odometry/filtered/global*). The package exposes the noise covariance matrices as configuration parameters to allow for tuning. Sensor variables were monitored, and their corresponding values in the covariance matrices were adjusted based on their convergence speed.

#### 2.3.2 Path generation and modified pure pursuit path tracking

All data collection and experiments for this study were conducted at the UGA Tifton Tifton Campus fields located at (31.471987 N, 83.527951 W) in Tifton, GA.

The paths between cotton rows are represented by pre-recorded GPS points where the robot was driven in the field. The GPS points were converted to UTM (Universal Transverse Mercator) format which represent the latitude and longitude values in meters instead of degrees. To get smooth paths the UTM points were connected using cubic spline interpolation method (McKinley and Levine, 1998). This method utilizes a third-degree polynomial to connect points and generate other points in between, which enabled us to generate points with a resolution of 3 cm. The cubic spline interpolation method was preferred to other alternatives due to its lower complexity in its implementation on top of its smoothness and small error (Press et al., 2007; Chewi et al., 2021).

**FIGURE 3:**
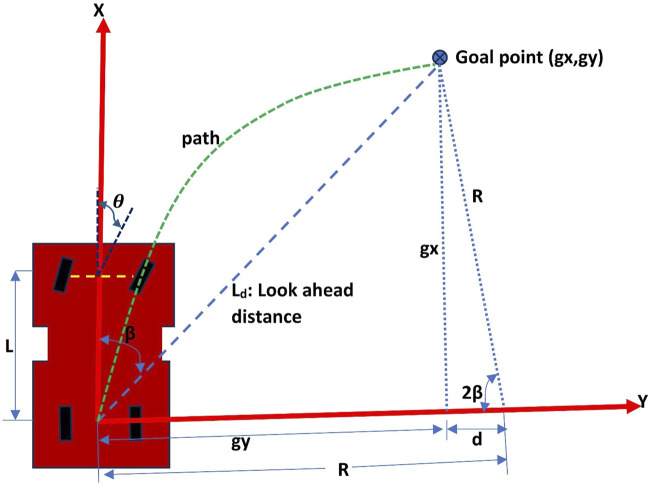
Pure pursuit mechanism. θ is the steering angle, $L_{d}$ is the look ahead distance, L is the wheelbase, and β is angle between the robot’s heading and the look-ahead line.

After a path is generated, the robot needs to follow that path through tracking its own pose and determine the steering angle needed to remain on the desired path. One of the well-known path-tracking algorithms is pure pursuit. Pure pursuit (Coulter, 1992; Park et al., 2014; Wang et al., 2017; Fue et al., 2020) is a geometric path tracking controller. It follows a look-ahead target point at a fixed distance on the reference path. The target point (Goal point as shown in Figure 3) along the desired path is selected at a distance $L_{d}\;\left(m\right)$ from the position of the rover which is taken at the center of the rear axle.

With the aim of making the robot steer at a correct angle $\theta \;\left(rad\right)$ to get to the target point, the geometric relationships were calculated. Considering the angle between the robot’s heading and the look-ahead line as $\beta \;\left(rad\right)$ , the robot traverses a curved path of radius $R\;\left(m\right)$ . To make the curved path unique, its center is chosen to lie on the y-axis forming a curve with radius R . From Figure 3, the relationships are defined below:

$$R=\left|gy\right|+d$$

$$d^{2}+{gx}^{2}=R^{2}$$

$${gx}^{2}+{gy}^{2}={L_{d}}^{2}$$

Solving these equations, results into,

$$R=\frac{{L_{d}}^{2}}{2\times \left|gy\right|}$$

But,

$$\sin\left(\beta \right)=\frac{\left|gy\right|}{L_{d}}$$

So,

$$R=\frac{L_{d}}{2\sin\;\left(\beta \right)}$$

From the robot kinematics, the relationship between R , wheelbase L , and the steering angle θ ,

$$R=\frac{L}{\tan\;\left(\theta \right)}$$

So, the steering angle θ can be calculated as:

$$\theta =\arctan\;\left(\frac{2Lsin\left(\beta \right)}{L_{d}}\right)$$

The pure pursuit controller ignores dynamic forces on the vehicle and assumes constant speed, however, at shorter look-ahead distances, the controller would be dangerously aggressive at high speeds which lead to instability. So, the algorithm was modified to set the robot’s speed depending on the steering angle calculated, the target rover speed $V_{T}\;\left(\frac{m}{s}\right)$ is set inversely proportional to the steering angle θ by a gain $K_{s}$ .

$$V_{T}=K_{s}\times \theta$$

#### 2.3.3 Speed control using PID

Since the field surface is uneven and contains some washed out gullies, maintaining velocity of the rover is challenging. A PID controller (Proportional, Integral, and Derivative) (Ang et al., 2005; Wang, 2020) was used for speed control. A PID controller continuously computes the difference between a desired setpoint value and a measured variable, then applies correction on the control value based on three pre-tuned gains, proportional, integral, and derivative. For speed control, the control value at time t , $u\left(t\right)$ is the motor command. Given the current velocity of the robot $V\left(t\right)\;\left(\frac{m}{s}\right)$ , the PID system calculates the motor command required by the robot to reach the desired targeted rover velocity $V_{T}\left(t\right)\left(\frac{m}{s}\right)$ , using the three gains, proportional gain $K_{p}$ , integral gain $K_{i}$ , and derivative gain $K_{d}$ .

The error value at time t , $e\left(t\right)$ is given by:

$$e\left(t\right)=V_{T}\left(t\right)-V\left(t\right)$$

The motor command PID value $u\left(t\right)$ is obtained from:

$$u\left(t\right)=K_{p}e\left(t\right)+K_{i}\int_{0}^{t}e\left(\tau \right)d\tau +K_{d}\frac{de\left(t\right)}{dt}$$

The overall motor command is a combination of the open-loop control value $z\left(t\right)$ , representing the ideal motor command to drive the rover at target speed, and the PID output $u\left(t\right)$ , which compensates for the remaining error between the current rover velocity and the target velocity.

The PID controller was tuned to find the values of the three gains that achieved desired performance, by iteratively adjusting the gains while monitoring the step response of the rover. The time step was set at 50 ms.

**FIGURE 4:**
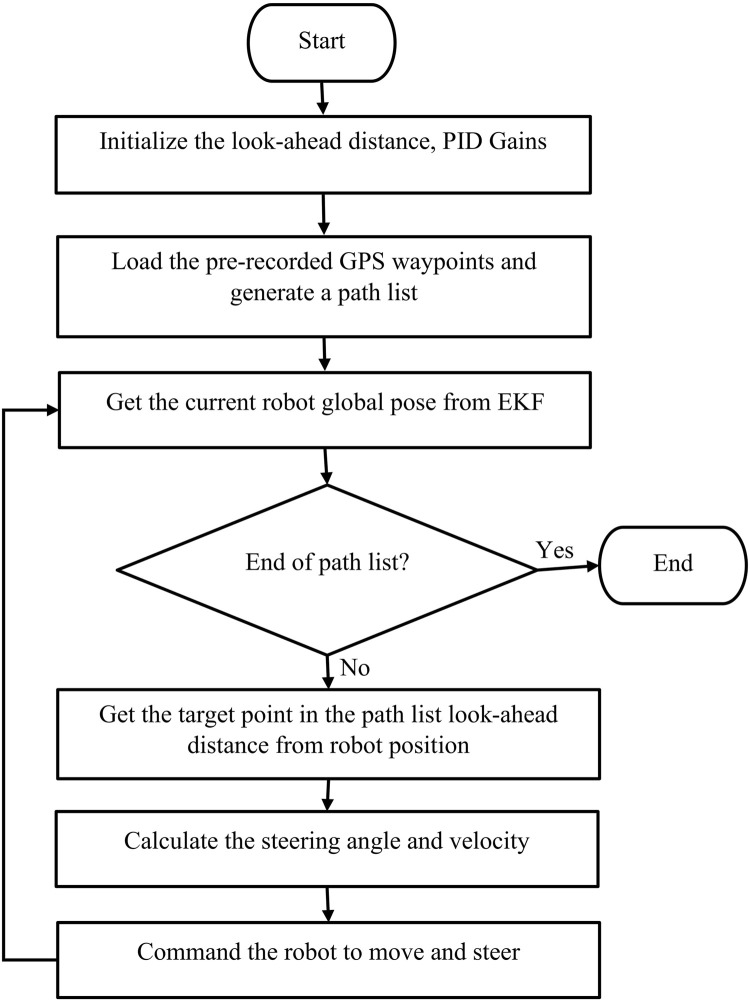
Schematic workflow of autonomous navigation GPS navigation.

The overall navigation process using pursuit is summarized in Figure 4.

### 2.4 Visual navigation using deep learning

Unlike GPS-based navigation systems, visual navigation systems are robust to interference, high resolution, and low cost (Leordeanu and Paraicu, 2021; Arafat et al., 2023). With vision, obstacle avoidance can also be implemented. Vision technology can be utilized to detect paths or crop rows in the field.

#### 2.4.1 Path detection using fully convolutional network (FCN) for semantic segmentation

**FIGURE 5:**
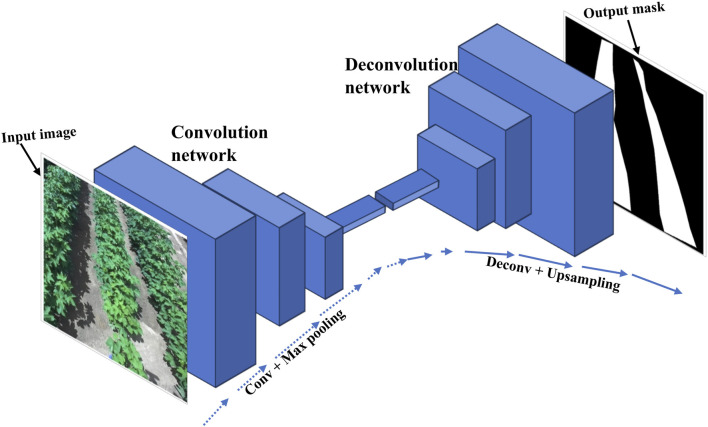
A fully convolutional neural network structure with input image and output segmentation mask.

FCN for semantic segmentation, Figure 5, classifies each pixel in an image to a predefined class. The network consists of two parts. The first part is the convolution/encoder network which uses an object classification network such as VGG Net (Simonyan and Zisserman, 2015), as a backbone to shrink the spatial resolution of feature maps in the image and detect the important features. The second part is the deconvolutional/decoder network which up-samples using bilinear interpolation and increases the spatial resolution of the features to classify each pixel from the original image into a class. The input to the FCN is an image and the output is another image (a segmentation mask) which has the same size as an input image with each pixel representing a predefined class.

For path detection, the image of cotton rows (Figure 5 input) is fed into the network, and the network predicts the segmentation mask as shown in Figure 5 output, with the white pixels representing the path between the rows.

##### 2.4.1.1 Model creation

Cotton rows images (resolution 1,280 × 720 pixels) were acquired from the field using a ZED2 stereo camera mounted on a rover at different cotton growth stages up to 40 days after emergence, times of the day, weed densities, shadows, and camera angles. Normally, cultivated cotton plants take nearly 6 months to reach harvest, progressing through different growth stages such as seed germination, seedling, vegetative growth, flowering, and boll development (Ritchie et al., 2007). Thriving cotton plants can grow up to 1 foot per month, reaching heights of up to 5 feet before harvest.

More than 400 images were labeled to indicate the path between cotton rows using Label Studio software (https://labelstud.io/). Since only paths were considered, there was only one segmentation class. The impact of crop shadows on segmentation was addressed by incorporating a diverse range of images within the training dataset. This included images with and without shadows, allowing the model to learn path characteristics while remaining robust to shadows encountered in real-world field conditions. The ground truth labels for the dataset focused solely on identifying the path between cotton rows, regardless of shadows present in the image. Furthermore, to address the challenges of varying environmental conditions, our dataset incorporated images capturing a diverse range of factors such as images with different lighting conditions, weed densities and different growth stages up to 40 days after emergence as this period is most susceptible to weed interference and aligning with the project’s focus on autonomous weeding. There are about 3 growth stages from the seedling emergence to about 40 days. These stages were captured in the dataset for training. On weeds concerns, the presence of weeds, especially during early growth stages when some varieties resemble cotton plants, posed a challenge for FCN performance. To mitigate this, the training dataset was specifically enriched with images containing weeds to enable the model to learn and differentiate between weeds and cotton. The labeled dataset was randomly split into training dataset (80%), validation dataset (10%), and testing dataset (10%). The FCN segmentation model was trained on a training dataset while being validated on a validation dataset. The model was trained on a deep learning computer (32–cores Intel I9, 2 Nvidia RTX 2080 GPUs, 128 GB RAM). After training, the model was run on the testing dataset to test its performance.

#### 2.4.2 Robot navigation

**FIGURE 6:**
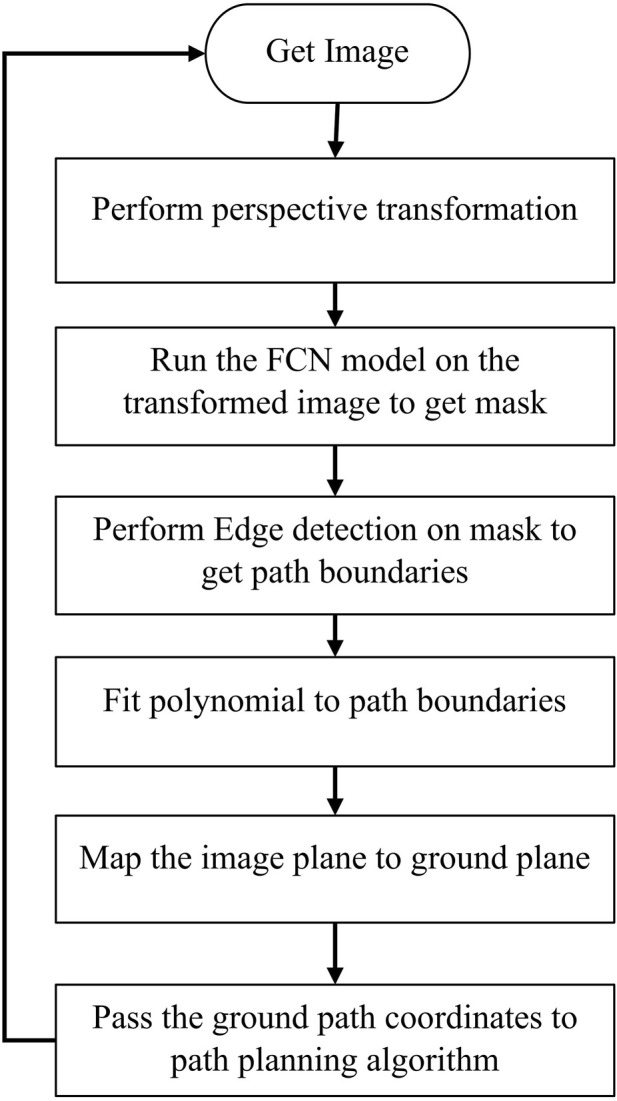
Context of the process to detect path and map to ground coordinates.

The FCN model predicts the path between the cotton rows and outputs the segmentation mask which is just physical pixels in the image domain that represents the path. For the rover to navigate, its position on the ground relative to the detected path must be known together with the next target coordinates, so the detected path in the image plane must be mapped to the ground plane. Figure 6 shows this process.

**FIGURE 7:**
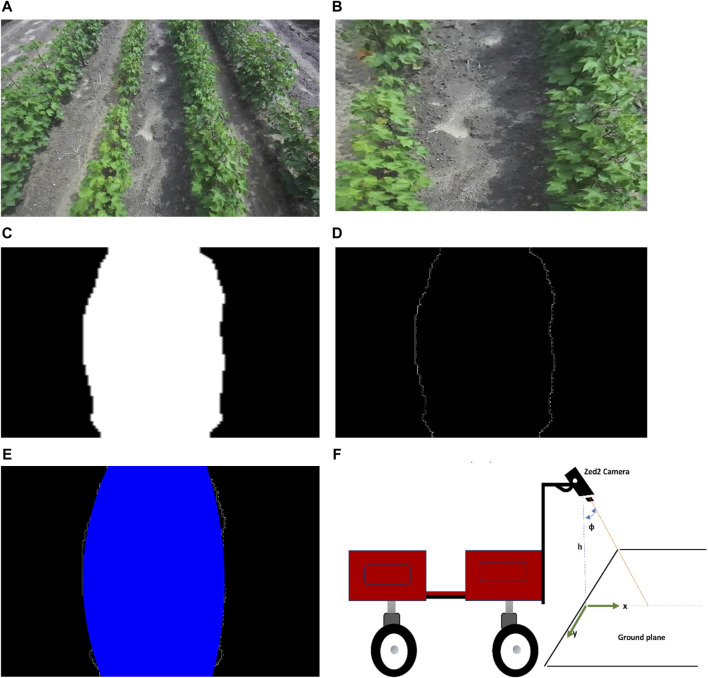
Detecting and mapping path between rows. **(A)** Acquired image from front camera, **(B)** Bird’s eye view of the image, **(C)** A segmentation mask of the transformed image, **(D)** Edge detection result on the segmentation mask, **(E)** Polynomial fitted into left and right path boundaries, **(F)** Camera setup with respect to ground plane.

The image from the ZED2 stereo camera in front of the rover (Figure 7A) was acquired. The camera was mounted at a height of 1.5 m from the ground and inclined at an angle of 25° from the vertical axis. The left image sensor of the ZED2 stereo camera was used as a reference axis, the resolution of the image was 1280 × 720 pixels. ZED2 has a field of view (FOV) of 110° (H) × 70° (V) × 120° (D).

From camera perspective, parallel lines appear to converge further away from the camera lens which make the path between cotton rows appear narrower away from the camera lens. To get accurate parallel lines, perspective transform of the image is required. Perspective-transform maps image points to new image points with a new perspective. The cotton rows image is transformed to a bird’s-eye view that represent the rows seen from above (Figure 7B) (only the path between the center two rows is targeted).

To detect the path, the FCN model was run on the transformed image to produce a segmentation mask (Figure 7C) with the white pixels representing the path in the image.

To find the path boundaries, edge detection was performed on the segmentation mask to get the pixels that represent the left and right boundaries of the detected path (Figure 7D). Canny edge detection algorithm (Canny, 1986) was applied to the mask to get the edges between path (white pixels).

To be able to map the path detected in the image domain to the ground plane, a plane representing the detected path in the image domain is needed. Since we already have the pixels representing the detected path boundaries, we fit polynomials to those pixels to get the left and right lines which form a plane (Figure 7E). A second order polynomial is fitted to the boundaries to account for the chance that the rows may be curved. While more sophisticated path mapping approaches exist, second-order polynomials proved to be a practical and effective solution. This is because most field paths consist of relatively straight lines with minor curves. This choice exemplifies the importance of striking a balance between complexity and practicality.

Since the camera FOV, resolution, and position with respect to the rover is known, the pixels in the image domain $\left(u,v\right)$ were mapped to the $\left(x,y\right)$ coordinates on the ground plane. Establishing our ground coordinates origin directly below the camera center (Figure 7F) with the camera height h meters from the ground, inclined at ∅ from the vertical axis, vertical FOV angle of θ , horizontal FOV angle of φ , and resolution $\left(H\times W\right)$ , the $\left(x,y\right)$ coordinates on the ground mapped from the $\left(u,v\right)$ pixel coordinates are given by the following equations:

x=h×tan∅−θ2+H−uH×tan∅+θ2−⁡tan∅−θ2

y=h×2v−WW×⁡tanφ2

#### 2.4.3 Path planning with dynamic window approach (DWA)

**FIGURE 8:**
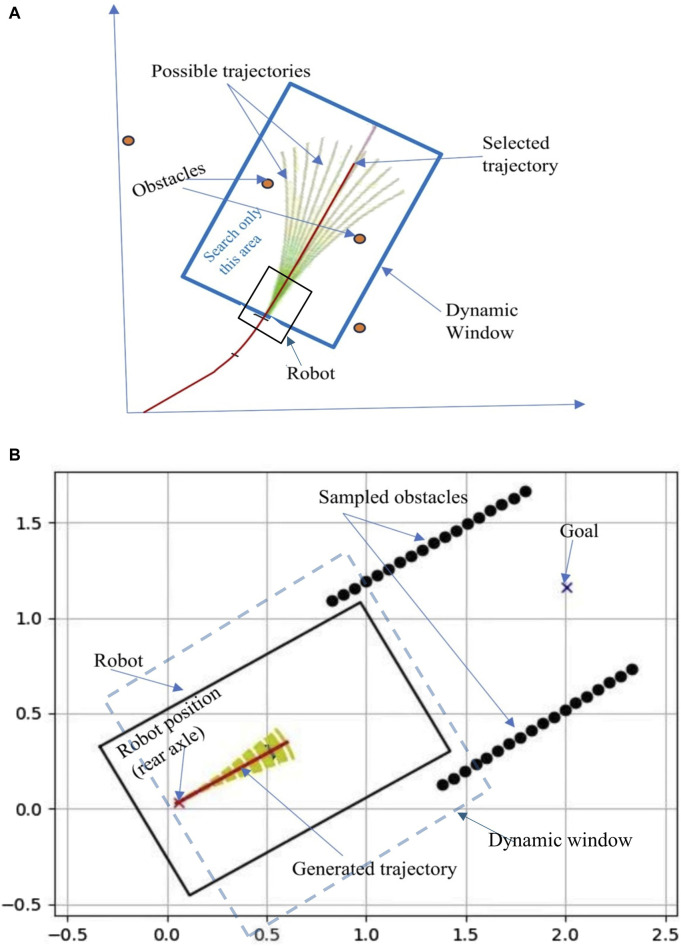
**(A)** Dynamic Window Approach, **(B)** Path boundaries points sampled to represent obstacles in DWA algorithm.

The DWA (Fox et al., 1997) is a velocity-based local path planning algorithm that tries to find the optimal collision-free velocities for the robot to navigate. This algorithm takes the robot’s kinematics into consideration when deciding a solution. Considering the limited accelerations of the motors, the search space for a solution is done in a ‘dynamic window’ which contains only the velocities that can be reached within the next time interval (Figure 8A). The algorithm works by generating trajectories determined by translational and rotational velocities (υ,ω), then selecting admissible velocities which avoid obstacles or can make the robot stop before it reaches an obstacle. The dynamic window created contains only the admissible velocities that can be reached within a short time interval given the limited dynamics of the robot.

Because of its obstacle avoidance nature, DWA can be used to navigate the robot between cotton rows and avoid running over the rows by treating them as obstacles. So, points along the detected path boundaries are sampled and fed to the algorithm as obstacles, then the generated trajectory avoids those points.

DWA considers three main parameters for optimization: The first parameter is Heading. It measures how close the robot’s direction is to the targeted goal location. The algorithm prefers the direction that moves the robot closer to the goal. Another parameter is Obstacle distance, which measures the distance to the closest obstacle on the trajectory, smaller distances increase the robot’s desire to move around them. The third parameter is the forward velocity of the robot, the algorithm tries to choose the highest admissible velocity for fast movement. In general, the algorithm finds a set of linear and angular velocities $\left(v,\omega \right)$ that optimize the objective function $F\left(v,w\right)$ containing heading with gain α , obstacle distance with gain β , and velocity with gain γ .

$$F\left(v,w\right)=\delta \left(\alpha .heading\left(v,\omega \right)+\beta .obstacle\left(v,\omega \right)+\gamma .velocity\left(v,\omega \right)\right)$$

Navigation in the cotton field using DWA was done by continuously giving the algorithm a goal point which is a point along the center line (2.0 m ahead of the robot position) of the predicted path by FCN network. The obstacles were represented by the sampled points on the path boundaries as seen in Figure 8B.

### 2.5 GPS and FCN network navigation combination

The FCN network navigation has the advantage of stability and since the path is observed in real-time, the robot can plan to account for any dynamic changes in real-time while navigating between the rows. However, it does not know the overall map of the field which can be challenging especially when the robot is turning to go to another row. Global path planning is ideal for this situation. Despite its shortcomings, GPS can easily map the entire field. A better solution is to leverage the advantages of both GPS and deep learning by combining them. This solution is a novel idea proposed in this study. GPS can be used for global planning to map the entire field, while deep learning acts as a local planner, to detect paths between the rows in real-time, navigate and avoid obstacles. So, the robot follows the GPS global path while adjusting its movement based on the visual observation.

**FIGURE 9:**
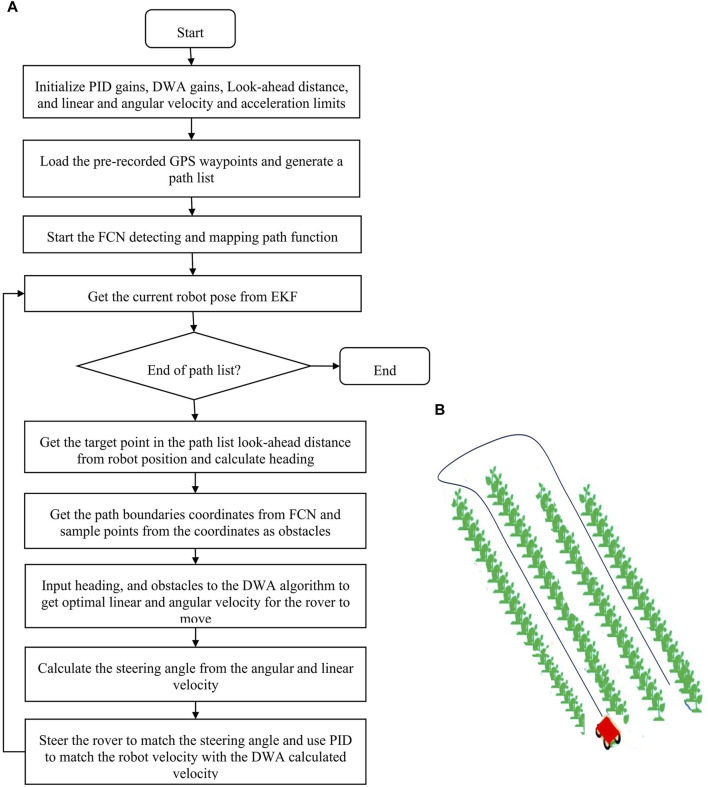
**(A)** Shematic workflow of autonomous navigation using a combination of GPS and visual navigation (GPS, FCN, and DWA), **(B)** Robot’s movement pattern in the field.

This solution was implemented by getting the target goals from pre-recorded GPS coordinates, using FCN to detect the path between the rows, then finding the velocities ( v,ω ) that approach the goal but not collide with obstacles. The schematic workflow of the solution is shown in Figure 9A.

### 2.6 Experiments

To test the solutions, experiments were conducted in the fields. The field layout consisted of 48 plots, each with four rows. To navigate the entire field, the robot followed a specific pattern. It would begin on the path between the first and second rows. At each turn, due to its turning radius limitations, it would skip one row (e.g., avoid the path between the second and third rows). Upon reaching the end of the field, it would then return to the first skipped path and repeat the pattern. This ensured that all rows were eventually covered. For GPS navigation experiments, the robot was run manually first between rows of cotton field, turn at the end and come back through another pair of rows, skipping one row as in Figure 9B, while recording the GPS coordinates to form pre-recorded way-points. Then the robot was autonomously driven following the recorded pattern using pure pursuit. This was repeated four times. The robot’s GPS position was recorded while navigating for comparison with the path followed. For FCN and DWA experiments, the fully convolutional neural network for semantic segmentation model was tested on testing dataset for its accuracy in detecting the paths between the rows. Then, in the cotton field, utilizing the model to detect path and DWA to navigate, the robot was run four times between cotton rows. The ideal path (center line between the rows) and the robot’s position was recorded for comparisons. And finally, visual navigation (using FCN) and GPS were combined and tested. Using the pre-recorded GPS coordinates from the GPS experiment as the desired path, the rover navigated between the rows following the coordinates while restricting its movement based on the path detection information and DWA planning. The combination of GPS and visual navigation serves as a crucial redundancy measure. If one system encounters temporary issues due to environmental changes, the other can maintain navigation until normal operation is restored, for example, when the robot does not detect the path, at the end of the rows, the robot uses GPS only solution to navigate until it detects the path again.

### 2.7 Evaluation metrics

The FCN network for semantic segmentation model was evaluated using Pixel Accuracy like in Long et al. (2015), Intersection over Union (Jaccard Index), F1 score (Dice coefficient), Precision, and Recall like in Torres et al. (2020). It was also evaluated on inference time, and number of frames per second (fps) metrics.

Pixel accuracy represents the percentage of pixels in the image that are classified correctly.

Precision measures how well the positive predictions match the ground truth.

$$Precision=\frac{True\;positives}{True\;positives+False\;positives}$$

Recall measures how many relevant predictions are made out of all predictions.

$$Recall=\frac{True\;positives}{True\;positives+False\;negatives}$$

Intersection over Union (IoU) indicates the overlap of the predicted bounding box coordinates to the ground truth box.

F1 score is calculated from precision and recall representing the model’s accuracy.

$$F1\;score=2\frac{Precision\times Recall}{Precision+Recall}$$

Inference time measures the time it takes for a model to make a prediction on a single image, and frames per second indicate the frequency at which inference is performed on consecutive images in a video stream. The inference time and fps were tested on the robot’s embedded computer (Nvidia Jetson Xavier AGX) for real-field experience.

To evaluate the accuracy of the robot’s path following ability, a trajectory similarity measure was used. This method samples points along the path and calculates the lateral distance error between the desired path and the path generated by robot’s movement. The points along the path were sampled at 0.05 m distances, then the lateral error was evaluated.

## 3 Results

### 3.1 FCN model accuracy

**FIGURE 10:**
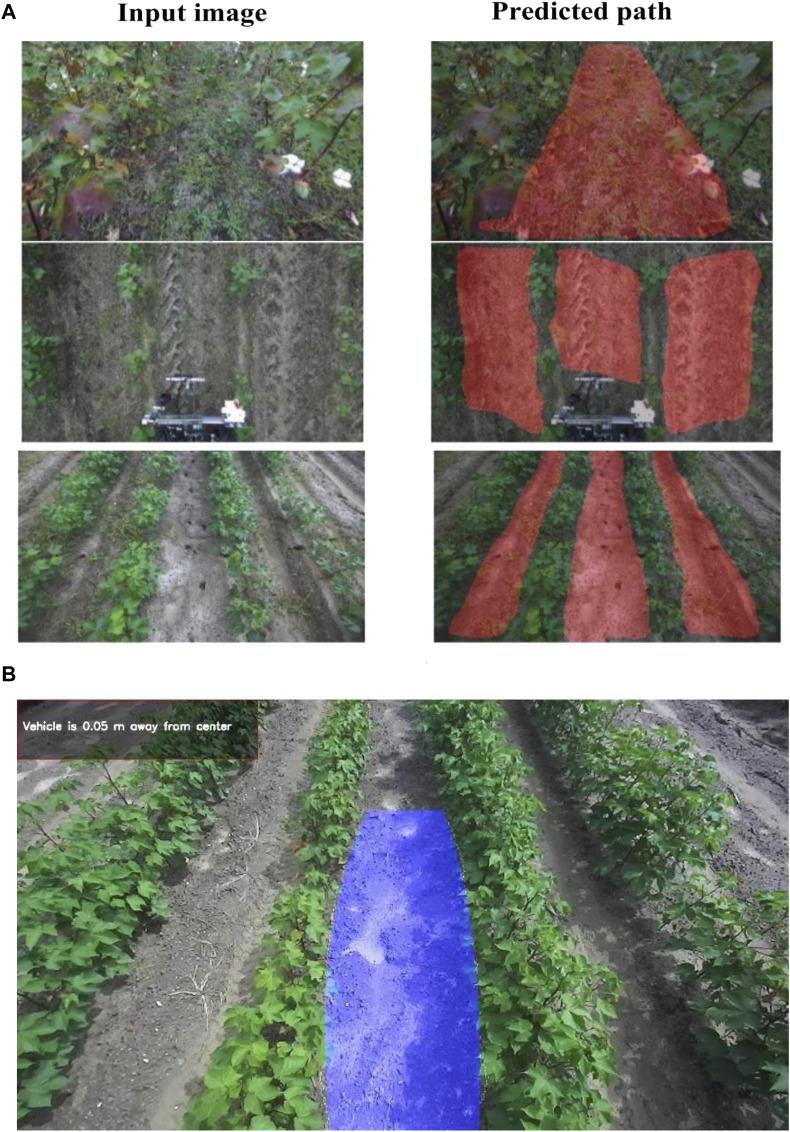
**(A)** Examples of tested images on the FCN model and their predicted results, **(B)** Path detected between cotton rows and mapped to ground plane.

Table 1 shows the model performance results when it was evaluated on testing dataset. The model performed well with a pixel accuracy of 93.5%, F1 score of 87.8%, and IoU of 79.5%. However, the model was relatively slow on robot’s embedded computer which attained a reference time of 182 ms and five fps. Visual observation (Figure 10A) showed that the model was able to detect paths between cotton rows and was robust to camera angles, cotton growth stages, weeds, and occlusions in the field. The process to map detected path in image domain to ground coordinates worked when tested on field images, the algorithm isolated a single row, found a path and mapped it into ground coordinates as in Figure 10B.

**TABLE 1:** FCN model performance evaluation results.

Metric	Value
Pixel Accuracy	0.935
Intersection over union	0.795
Precision	0.908
Recall	0.859
F1 score	0.878
Inference time (ms)–embedded computer	182
Frames per second (fps)–embedded computer	5

### 3.2 Navigation experiments

#### 3.2.1 GPS navigation

**FIGURE 11:**
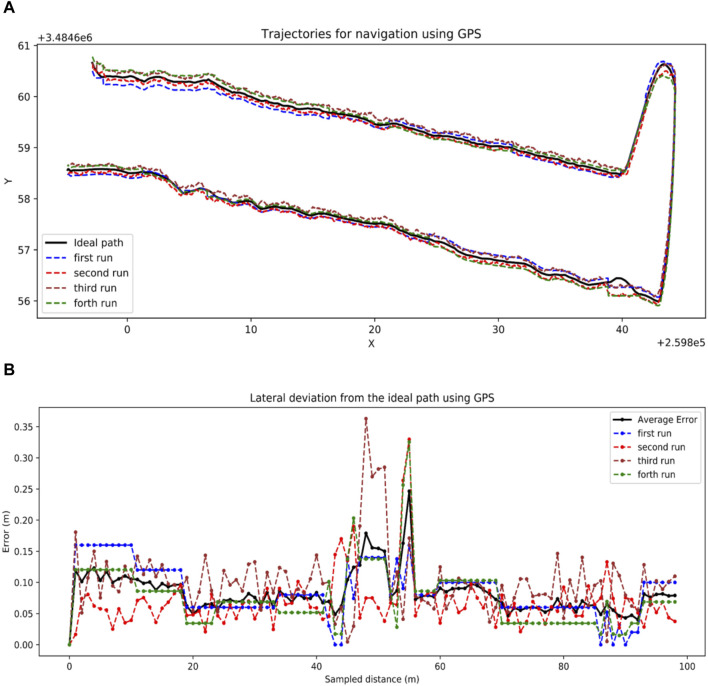
GPS navigation results. **(A)** Trajectories generated by pure pursuit following GPS path, **(B)** Lateral distance error of the paths generated by the robot using GPS and pure pursuit.

The robot was able to follow the pre-recorded GPS coordinates in the field using pure pursuit path tracking algorithm (Figure 11A). The shape of the trajectory at end-of-row turning is due to the large turn radius of the robot as it tries to go to the next plot. There were slight deviations when the robot was turning, which is demonstrated by the deviation errors in Figure 11B. The lateral deviation errors for trials seem to peak around turning locations. The average lateral deviation was 8.3 cm which is good for navigating the field.

#### 3.2.2 Visual navigation

**FIGURE 12:**
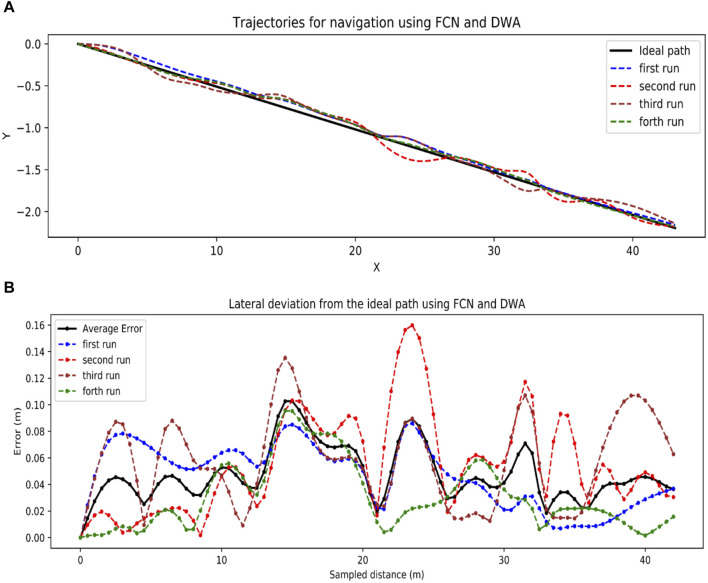
Visual navigation results. **(A)** Trajectories generated using FCN and DWA, **(B)** Lateral distance errors by FCN and DWA.

Using DWA and deep learning, the robot was able to follow the ideal straight path (center line between the rows), as demonstrated in Figure 12A, with an impressive average lateral deviation of 4.8 cm as in Figure 12B. Despite the low lateral deviation, this solution lacked a mechanism for turning at row ends, hindering its ability to transition to subsequent rows.

#### 3.2.3 GPS and visual combination

**FIGURE 13:**
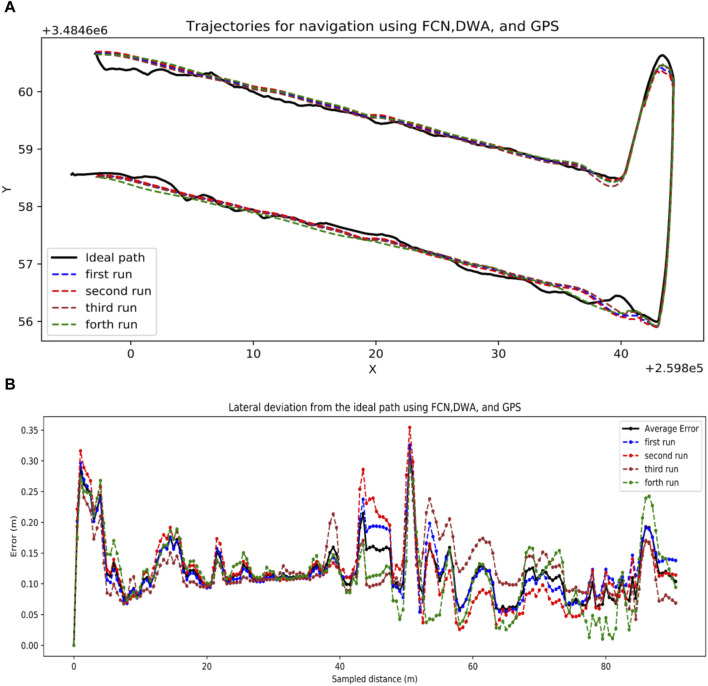
GPS and Visual combination results. **(A)** Trajectories generated by a combination of FCN, DWA and GPS, **(B)** Lateral distance deviations by combining FCN, DWA, and GPS.

With a combination of GPS and FCN, the robot successfully followed the GPS coordinates as a global path while performing path detection and avoided obstacles (rows). It can be observed in Figure 13A, the path profiles for all the runs look the same because they were trying to go straight forced by the DWA local planner while the GPS coordinates were not as straight. The average lateral deviation was 12.1 cm which was higher than the other solutions, however, it was skewed at the turning locations as shown in Figure 13B, and by the pre-recorded GPS points not making a smooth straight path. The average deviation of row following separately was 9.5 cm, while that of end-of-row turning was 14.6 cm. This was still a more practical choice for real-world field navigation since it combines the strengths of both GPS and visual navigation, achieving a balance between accuracy and robustness through effective row navigation, end-of-row turning mechanism, maintaining navigation even when one sensor fails, and the potential for obstacle avoidance.

## 4 Discussion

Autonomous navigation in cotton fields using GPS and visual navigation techniques was successfully implemented and tested in a cotton field. In GPS navigation, a pure pursuit path tracking algorithm was used to follow pre-recorded GPS coordinates in the field. The solution was able to follow the path generated by the coordinates with an average lateral distance error of 8.3 cm. In visual navigation a deep learning model, fully convolutional neural network for semantic segmentation model was trained to detect paths between cotton rows, then a local path planning algorithm, dynamic window approach (DWA) used the detected path to navigate the robot. The model achieved a pixel accuracy of 93.5%, F1 score of 87.8%, and five frames per second speed on an embedded computer used by the robot. Moreover, the detected paths were successfully mapped to the ground coordinates for robot navigation. Field testing demonstrated successful autonomous navigation between cotton rows using the dynamic window approach path planning technique, achieving an average lateral deviation of only 4.8 cm from the desired path. However, while effective within-row navigation was achieved, the solution lacked a mechanism for turning at row ends to transition to the next row. This limitation could be addressed by incorporating a pre-defined turning pattern upon end-of-row detection. However, such an approach might not be robust or guarantee successful transition to the next path.

By combining the strengths of each solution, GPS with its global mapping ability, and visual navigation with its ability to detect paths in real-time and avoid obstacles, a new solution was implemented. GPS provides global path planning by mapping the field, while visual navigation and the Dynamic Window Approach (DWA) handle local path planning, navigation, and real-time decision-making. This combined approach achieved an average lateral deviation of 9.5 cm while following rows and 14.6 cm during end-of-row turns. Several factors make this solution desirable for real-world field navigation: Firstly, its effectiveness in navigating the rover between the crop rows. Secondly, its end-of-row path completion which means the robot can follow the path even at the end of rows, enabling autonomous navigation of the entire field using pre-recorded path plans. Moreover, the solution provides sensor redundancy, implying that the system maintains navigation even if one sensor fails. This is crucial, as GPS signals can be lost, and the visual component (FCN) may occasionally struggle to detect the path. Another advantage of this solution is its potential for real-time obstacle avoidance. The inclusion of visual navigation offers the potential for real-time obstacle avoidance, minimizing collisions with animals, humans, or other objects during autonomous. Overall, this combined solution leverages the unique capabilities of both GPS and visual navigation, resulting in a more robust and adaptable system for autonomous agricultural robots operating in real-world field conditions.

Several key challenges were identified during the study that impacted the results. The primary challenge stemmed from the inherent uncertainties and complexities of the agricultural environment. Visual navigation, in particular, was significantly affected by sudden changes in lighting, weather, crop growth, weed emergence, and shadows. While the FCN performed well, it occasionally encountered unforeseen scenarios, leading to errors. Additionally, rough terrain made velocity control more difficult, hindering the effectiveness of the PID controller. GPS data inconsistencies also impacted navigation, preventing the robot from following a precise, straight line.

Potential improvements that can be explored in future studies to address these challenges include: enhanced training data diversity which will enable training the FCN with a wide range of data encompassing various field conditions to improve its ability to handle unforeseen scenarios, Adaptable path mapping methods utilizing machine learning-based methods, spline interpolation, and hybrid approaches to enhance robustness and accuracy of path mapping, advanced speed control mechanisms which involve implementing alternative speed control methods such as Model Predictive Control (MPC) to improve responsiveness and performance on rough terrain, and 3D camera integration by leveraging the depth information from the stereo camera, this can potentially improve navigation by providing a more comprehensive understanding of the environment.

## Data Availability

The raw data supporting the conclusion of this article will be made available by the authors, without undue reservation.
